# The Role of Interleukin 17 in Association with Systemic Parameters in Severity Stratification and Mortality Prediction in Moderate and Severe Acute Pancreatitis

**DOI:** 10.3390/medsci14060600

**Published:** 2026-09-23

**Authors:** Diana Iosif, Adrian Paul Suceveanu, Alina Doina Nicoara, Cristian Ionut Orasanu, Marius Dragos Prazaru, Iulia Cindea, Viorel Gherghina, Florin Ciobanu, Anca Chisoi, Miruna-Gabriela Vizireanu, Anca Pantea Stoian, Adrian Costache, Andra Iulia Suceveanu

**Affiliations:** 1 Doctoral School of Medicine, “Ovidius” University of Constanta, 900470 Constanta, Romania; 2 Department of Intensive Care Unit, “Sf. Apostol Andrei” Emergency County Hospital, 900591 Constanta, Romania; 3 Faculty of Medicine, “Ovidius” University of Constanta, 900470 Constanta, Romania; 4 Department of Gastroenterology, “Sf. Apostol Andrei” Emergency County Hospital, 900591 Constanta, Romania; 5 Clinical Service of Pathology, Department of Pathology, “Sf. Apostol Andrei” Emergency County Hospital, 900591 Constanta, Romania; 6 Center for Research and Development of the Morphological and Genetic Studies of Malignant Pathology (CEDMOG), “Ovidius” University of Constanta, 900591 Constanta, Romania; 7 Department of Diabetes, Nutrition and Metabolic Diseases, “Carol Davila” University of Medicine and Pharmacy, 020021 Bucharest, Romania; 8 Department of Diabetes and Metabolic Diseases, “Prof. N.C. Paulescu” National Institute of Diabetes, Nutrition and Metabolic Diseases, 020475 Bucharest, Romania; 9 Department of Pathology, “Carol Davila” University of Medicine and Pharmacy, 020021 Bucharest, Romania; 10 Ultrasound Teaching Unit, “Dr. I. Cantacuzino” Clinical Hospital, 030167 Bucharest, Romania

**Keywords:** acute pancreatitis, interleukins, severe acute pancreatitis, risk

## Abstract

**Introduction:** Acute pancreatitis is an inflammatory lesion that can lead to multiple organ dysfunctions and has an increased mortality rate. The pathogenic mechanisms are complex. A central element is represented by systemic inflammation. This exploratory study aims to identify parameters useful in stratifying the severity of moderate and severe acute pancreatitis and predicting mortality, as well as in evaluating the predictive value of IL-17 regarding disease severity. **Material and Methods:** A retrospective study was conducted during the period October 2024–August 2025 on an eligible group of 64 patients with moderate and severe acute pancreatitis. Demographic, etiological, and biological data were collected according to the observation sheets, anamnesis, and clinical examination. Also, the level of IL-17 was quantified by the ELISA technique. All data were appropriately analyzed statistically. **Results:** Of the 64 cases, 35 (54.69%) had severe acute pancreatitis. From the clinical–demographic data, we identified personal cardiovascular and renal pathological antecedents as independent risk factors for death (OR = 7.529 and OR = 24, respectively). Also, metabolic etiology represented a risk factor for death (OR = 5.625). From the biological parameters, erythrocyte sedimentation rate ≥ 41.29 mm/h and AISI score ≥ 1627.50 have a role in predicting severity with quite high sensitivity and specificity. Leukocytosis, C-reactive protein (CRP), IL-17, and hypocalcemia represent predictive risk factors for the occurrence of severe acute pancreatitis (OR = 3.733, OR = 1.136, OR = 1.068, and OR = 3.542, respectively). Of these, only CRP, IL-17, and hypocalcemia represent risk factors for death (OR = 1.051, OR = 1.080, OR = 6.771). Regarding the in-hospital management of intensive care days, complications and organ dysfunctions are risk factors for death. **Conclusions:** This study identified useful parameters adjunct to the current scoring systems to stratify patient severity in moderate and severe acute pancreatitis, as well as risk factors for mortality. In addition, IL-17 provides useful information involved in the pathogenesis, evolution of severity and survival in these patients. Despite the promising role of IL-17, these findings remain preliminary due to the small sample size and require external validation.

## 1. Introduction

Acute pancreatitis is an inflammatory lesion of the exocrine pancreas that can lead to multiple organ dysfunctions and progress to persistent organ failure and pancreatic necrosis [1]. It is one of the most common causes of hospitalization in the gastroenterological field. It has an incidence of 3.8–74.8 cases per 100,000 in Europe and 34 cases per 100,000 globally [2,3]. In Europe and North America, increases in its frequency by 3% have been observed due to the increased prevalence of risk factors but also to the improvement of detection techniques [4]. Even though the mortality rate has decreased from 1.6% to 0.8% of cases, it is the inflammatory disease with the highest mortality, which can reach 30–40% in severe cases [3,5].

Regarding etiology, the most common risk factors involved in the development of the condition are represented by gallstones and alcohol consumption (approximately 30–50% of cases each). Even though acute pancreatitis does not have a gender-specific predisposition, it has been observed that the biliary etiology is more frequently encountered in women and the ethanolic etiology in men. Other factors implicated in the production of acute pancreatitis are represented by hypertriglyceridemia (approximately 10% of cases), iatrogenic causes (5% of endoscopic retrograde cholangiopancreatography results in severe pancreatitis), and traumatic or rare causes (drugs, autoimmune diseases, genetic predispositions, or infections) [6].

A positive diagnosis includes at least two of the following criteria: lipasemia or amylasemia three times the normal value, specific clinical presentation (upper abdominal pain radiating to the back, vomiting, or nausea), or imaging criteria [2]. However, the disease is very complex. Despite clinical, biochemical, and imaging criteria for establishing the diagnosis and assessing severity, it is not an easy task to predict its subsequent evolution. The main reason is that the pathogenic mechanisms are not fully elucidated and need to be studied further [7].

The core of the pathogenic process is represented by systemic inflammation through early activation of proteases and the nuclear factor kappa-light-chain-enhancer of activated B cells (NFκBs). The first step is NFκB activation (in the first minutes), which has a role in regulating the innate and adaptive immune response, as well as leukocyte activation [8]. The innate immune system response recognizes the molecular aspects secreted by necrotic pancreatic cells and triggers an adaptive immune response [9]. T lymphocytes (helper, regulatory, or cytotoxic) serve as the primary effector cells of the adaptive immune response [10]. Through the secretion of cytokines, the initiation of the inflammatory response occurs. At the macro scale, the inflammatory response is responsible for organ malfunction, maintenance of tissue destruction, and, in severe cases, organ dysfunction. At the micro scale, it is responsible for the recruitment of inflammatory cells with the production of pro-inflammatory mediators (tumor necrosis factor-α—TNF-α and interleukins—IL-1β, IL-6, and IL-8) and anti-inflammatory mediators (transforming growth factor-β—TGFβ and interleukins—IL-4, IL-10, and IL-13) [11,12]. TNF-α is a central regulator of inflammation, leading to the activation of NFκBs [13]. Neutrophils and macrophages are the main ones responsible for the secretion of IL1β, which has a role in tissue destruction [11]. IL6 is involved in the IL6–Signal Transducer and Activator of Transcription 3 (STAT3)–Arid5a axis in immunological modulation [14]. IL8 is involved in the development processes of pancreatic fibrosis [11]. TGF-β can have a dual activity of promoting or progressing the disease. It has the role of inducing apoptotic phenomena and regulating the inflammatory process. Activating T lymphocytes, specifically differentiating Th9 and Th17 cells with pro-inflammatory activity, plays a crucial role in the inflammatory process [15]. IL4 and IL13 are produced by some of the Th2 CD4+ cells and have a role in the growth and differentiation of active B lymphocytes, as well as in the potentiation of the expression of class 2 antigens of the major histocompatibility complex [10,11]. The role is to modulate the activity of complement activation regulators involved in the occurrence of pancreatic necrosis. Neutralization of IL10 leads to an increase in the severity of pancreatitis and the amount of fibrosis [11].

The Th17/IL-17 axis plays a crucial pro-inflammatory role in acute pancreatitis. The production of granulocyte colony-stimulating factor and IL-8 is induced, promoting a massive influx of activated neutrophils into the pancreas and distant organs. In addition, IL-17A from pancreatic acinar and stellate cells stimulates the expression of inflammatory target genes, leading to the secretion of other cytokines (IL-1β, IL-6) and chemokines (CXCL1, CXCL2, CXCL5). These attract neutrophils and macrophages that locally destroy the pancreas and can produce acute respiratory distress syndrome systemically [10,11]. Activation and expansion of circulating Th17 cells, together with increased serum IL-17 concentrations, serve as significant prognostic markers that directly correlate with the development of early systemic inflammatory response syndrome and the severity of acute pancreatitis. We specifically selected IL-17 for investigation because of its critical position as a proposed upstream biological contributor to the inflammatory response. Unlike classical downstream biomarkers that accumulate in the bloodstream as a secondary consequence of structural tissue necrosis, the degree of activation of the Th17/IL-17 axis is thought to contribute to the secondary release of other cytokines. Therefore, measurement of IL-17 provides a potential early prognostic indicator that may reflect the immediate risk of severe systemic complications before extensive anatomical destruction occurs [10].

Given the complexity of pathogenesis and the involvement of systemic inflammation, the present study has two aims. The first is to identify new parameters useful both in stratifying the severity of pancreatitis (moderate versus severe) and in predicting mortality. The second aim is to evaluate the role of IL-17, in association with other clinical and paraclinical parameters, in view of its predictive role in the severity of the disease and its evolution towards the death of patients.

## 2. Materials and Methods

### 2.1. Study Design and Patient Selection

A retrospective study was conducted on patients admitted to the gastroenterology and intensive care units of the Emergency County Clinical Hospital in Constanta, Romania, between October 2024 and August 2025. Inclusion criteria included patients over 18 years of age who presented in the emergency room and received a diagnosis of moderate and severe acute pancreatitis. Exclusion criteria included minor patients, patients with incomplete laboratory or diagnostic data, and those admitted by referral or transfer from other medical facilities (Figure 1).

### 2.2. Clinical and Laboratory Evaluations

The diagnosis of severe acute pancreatitis was formulated based on local complications and/or persistent organ failure, for which the following criteria were used: Balthazar computed tomography score D or E, Ranson score ≥ 3, and APACHE II score ≥ 8. The IMRIE–Glasgow, BISAP, SOFA, and SAPS3 prediction scores were also used. The hospitalized patients benefited from the monitoring of laboratory biological markers daily and in case of status changes (hemoleukogram, aggregate index of systemic inflammation—AISI score, erythrocyte sedimentation rate—ESR, C-reactive protein—CRP, serum amylase, serum lipase, calcium, blood glucose, triglycerides, pH, bicarbonate, and lactate). However, all biological data presented in the Section 3 and in the tables strictly represent the initial blood sample at admission (obtained within the first 24 h of presentation, simultaneously with the IL-17 measurement), ensuring a standardized baseline for comparison. Demographic data (sex, age, environment) and etiological data (biliary, ethanolic, metabolic, post-interventional) were collected according to the observation sheets, the anamnesis (respiratory, cardiovascular, digestive, metabolic, neurological, or oncological comorbidities), and the clinical examination. Metabolic etiology refers strictly to a documented clinical history of class I-III obesity. The days of hospitalization, the days of intensive care admission, the complications that occurred (pancreatic abscesses, infected pancreatic necrosis, ascites, pleurisy), and the organ dysfunctions (pulmonary, cardiovascular, renal) were quantified.

### 2.3. Serum IL-17 Quantification

To assess IL-17 levels, peripheral blood samples were collected in sterile tubes by venipuncture immediately upon the patient’s admission to the Gastroenterology department (first blood sample), after his transfer from the Emergency Room, occurring uniformly within the first 24 h from symptom onset. Sample collection was performed after initial emergency stabilization (basic analgesia and oxygen therapy) but strictly before specific interventions at the ward level, such as fluid resuscitation, antibiotic administration, or transfer to the ICU. Serum was centrifuged at 1000× *g* for 20 min in a Thermo Scientific SL16R centrifuge (Thermo Fisher Scientific, Waltham, MA, USA). After extraction, serum samples were then divided into aliquots and stored at −80 °C until analysis. An IL-17 ELISA kit was used (ELISAKit ELK2610, ELK Biotechnology, Wuhan, China). The applied assay principle was the Sandwich immunoenzymatic assay. The microtiter plate was pre-coated with an IL-17-specific antibody. Samples were added to the corresponding wells of the microtiter plate, then with a biotin-conjugated antibody specific for IL-17. Avidin conjugated to horseradish peroxidase was added to each well and incubated. After the addition of the 3,3′,5,5′-tetramethylbenzidine (TMB) substrate solution, only the wells with IL-17, biotin-conjugated antibody, and enzyme-conjugated avidin showed a color change. The enzyme–substrate reaction was terminated by the addition of a sulfuric acid solution. Color changes were spectrophotometrically measured at a wavelength of 450 nm ± 10 nm. The concentration of IL-17 in the samples was determined by comparing the optical density of the samples with a standard curve made based on seven human IL-17 standards and the blank sample, with a detection limit and normal reference value established at <5.5 pg/mL for IL-17 in the general healthy population. The intra-assay and inter-assay precision was CV% < 8% and CV% < 10%, respectively. No significant cross-reactivity or interference with IL-17 fragment or analogs was observed.

### 2.4. Statistical Analysis

Statistical analysis was performed using SPSS Statistics Version 26 (IBM Corporation, Armonk, NY, USA). Indicators of central tendency and variability were used. Data normality was assessed using the Kolmogorov–Smirnov and Shapiro–Wilk tests, while the Levene test assessed homogeneity of variances. Given the non-normal distribution, variables were analyzed using the Mann–Whitney U test, the Kruskal–Wallis H test, and Fisher’s exact test. Continuous associations of the data were performed using the Spearman rank correlation. To evaluate the discriminatory performance of the parameters, receiver operating characteristic (ROC) curve and area under the curve (AUC) analyses were used. The optimal decision threshold (cut-off) was determined using the Youden index criterion, which maximizes the sum of sensitivity and specificity. Potential factors associated with clinical outcomes were evaluated strictly using univariate and multivariate logistic regression analyses, with results reported as odds ratios (ORs).

Precision of the parameters, the ROC curve and area under the curve (AUC) analysis were used. The optimal decision threshold (cut-off) for sensitivity and specificity was set at the value that maximizes the AUC value. Odds ratio (OR) assessment was performed by binary logistic regression or linear regression tests, depending on the nature of the data. The statistical significance threshold was set at a *p*-value ≤ 0.05.

### 2.5. Ethical Considerations

This study was conducted with approval by the Local Ethics Committee for Clinical Studies and Developmental Research, and written informed consent was obtained from all participants before the collection of biological samples (approval number: no. 46159, approval date: 11 August 2023).

## 3. Results

### 3.1. Clinical–Demographic Aspects

A group of 64 cases was identified, of which 23 (35.94%) were women and 41 (64.06%) were men. The median age of the group was 60 years (47–75.5 years). Of these, 35 (54.69%) had severe acute pancreatitis. Demographic and clinical data show a slight predominance of the male sex. Most patients had a personal pathological history, the most common being digestive, cardiovascular, and diabetes mellitus (Table 1).

Univariate and multivariate analyses of risk factors for death indicated a personal history of cardiovascular and renal pathology (Table 2, Supplementary Tables S1–S6).

The most common etiologies identified were biliary, ethanolic, metabolic, and post-interventional (Table 3). Metabolic etiology represented an independent risk factor for patient death (OR = 5.625, *p* = 0.022, CI95: 1.277–24.775).

### 3.2. Biological Aspects

Most patients had changes in biological markers (Table 4). An increased number of leukocytes was associated with increased IL-17 values (*p* < 0.001). An increased ESR value was associated with increased IL-17 values (*p* = 0.039). Increased AISI values were associated with increased IL-17 values (*p* = 0.008). Increased IL-17 values were associated with decreased serum amylase values (*p* = 0.026) and bicarbonate values (*p* = 0.005). Increased IL-17 values were associated with increased lactate values (*p* = 0.001). Hypocalcemia was observed in patients with a history of cardiovascular disease (*p* = 0.011). Hypocalcemia was correlated with leukocytosis (*p* = 0.002), an increased ESR (*p* = 0.028), and an increased CRP (*p* = 0.001). An ESR ≥ 41.29 mm/h has a sensitivity of 70% and specificity of 65% in predicting cases of severe pancreatitis (AUC = 0.726, *p* = 0.014). Also, an AISI score ≥ 1627.50 is predictive of severe acute pancreatitis with a sensitivity of 91.4% and a specificity of 75.9% (AUC = 0.924, *p* < 0.001). Patients with post-interventional etiology had low blood glucose values (*p* = 0.017), and those with metabolic etiology had high triglyceride values (*p* = 0.001).

Among the biological parameters performed, only leukocytosis, CRP, IL-17, and calcemia presented in the univariate analysis as risk factors for the prediction of severe acute pancreatitis. In the multivariate analysis, only CRP and IL-17 values represented risk factors (Table 5).

Univariate analysis of risk factors for death identified CRP, IL-17, and hypocalcemia, but only IL-17 showed statistical significance in multivariate analysis (Table 6).

An IL-17 value of ≥16.81 pg/mL is predictive of progression to severe acute pancreatitis (sensitivity of 77.1%, specificity of 55.2%, AUC = 0.649, *p* = 0.041), and a value of ≥19.23 pg/mL is predictive of death (sensitivity of 80%, specificity of 71.4%, AUC = 0.728, *p* = 0.008) (Table 7).

Seven diagnostic scores were used to highlight the correlations with the two degrees of severity of pancreatitis (Table 8). A statistically significant difference was observed between the CT score and the leukocyte count; the increased score correlated directly proportionally with the leukocyte count (*p* < 0.001). The same aspect was also found in the case of CRP (*p* < 0.001) and AISI (*p* < 0.001) values. A CT E score was statistically significantly correlated with low calcium levels (*p* = 0.023). The APACHE II score was directly proportionally associated with ESR (*p* = 0.012) and CRP (*p* = 0.009) values, and the RANSON score was associated with ESR level (*p* = 0.009). Increased IL-17 values were associated with increased RANSON (*p* = 0.020), IMRIE–Glasgow (*p* = 0.007), BISAP (*p* = 0.040), SOFA (*p* < 0.001), APACHE (*p* = 0.004), and SAPS3 (*p* = 0.014) scores.

### 3.3. Aspects Regarding Intrahospital Management

Patients with moderate acute pancreatitis had fewer days of hospitalization and intensive care, as well as a much lower death rate, compared to patients with severe acute pancreatitis (Table 9). The median duration of hospitalization was 7.5 days (IQR 4.25–12). Thirty (46.88%) patients were admitted to intensive care. Patients with an increased number of days of hospitalization also had an increased number of days in intensive care (*p* < 0.001). An increased number of days of hospitalization is justified by complications such as pleurisy (*p* = 0.001) or ascites (*p* = 0.005). Patients with an increased number of days in intensive care were associated with death (*p* < 0.001) and the need for vasoactive support (*p* < 0.001), as well as complications such as abscesses (*p* = 0.028), pleurisy (*p* = 0.011), ascites (*p* = 0.005), or ARDS (*p* = 0.020).

Patients who presented complications such as abscesses (*p* = 0.001), infected necrosis (*p* = 0.005), pleurisy (*p* = 0.012), and ascites (*p* = 0.019) were statistically associated with death. Death was also associated with the presence of ARDS (*p* < 0.001) and the need for vasoactive support (*p* < 0.001). The need for vasoactive support was associated with complications such as abscesses (*p* < 0.001), infected necrosis (*p* < 0.001), pleurisy (*p* < 0.001), and ascites (*p* = 0.044), as well as in the case of those with ARDS (*p* < 0.001).

Elevated IL-17 levels were associated with patients who died (*p* = 0.012), those who had infected necrosis (*p* = 0.008), those who needed vasoactive support (*p* = 0.004), and those with ARDS (*p* = 0.016). Patients with hypocalcemia had a high number of days of hospitalization (*p* = 0.028) and intensive care admission (*p* < 0.001). They also presented complications such as pleurisy (*p* = 0.005) and ascites (*p* = 0.005).

Univariate analyses of the aforementioned parameters identified numerous risk factors (Table 10), but without statistical significance in the multivariate analysis. Regarding dysfunctions, only cardiovascular dysfunctions showed statistical significance in the univariate and multivariate analyses (Table 11).

## 4. Discussion

The demographics of acute pancreatitis have seen an increase in incidence in recent decades. Li C. et al. observed an upward trend of acute pancreatitis, particularly among males [16]. The findings are supported by other studies, including the current one [17,18,19]. The median age of presentation, 60 years, is consistent with the literature, as is the fact that patients with severe acute pancreatitis are older [20,21]. It has been observed that after the age of 59, the number of severe cases increased significantly, as did the mortality of patients [20]. This aspect has made age an important parameter in the severity and mortality scores (APACHE II, Ranson, BISAP, and JSS) [22]. However, studies are divided regarding the risk factor for mortality generated by age. Some studies have reported that age is an independent risk factor for mortality, some have observed high age thresholds associated with mortality, and others have not observed these aspects [23,24].

Regarding comorbidities, we observed a major role in predicting death in patients with a personal history of cardiovascular and renal pathologies. The study conducted by Spagnolo DM et al. noted in patients with pancreatitis an increased influence of comorbidities in the digestive sphere, followed by cardiovascular ones (hypertension) [25]. Even though in our study, comorbidities in the digestive sphere were the most frequent, they did not impact survival. On the other hand, cardiovascular ones, dominated by arterial hypertension, did. One explanation may be the fact that this syndrome has multiple etiologies in common with pancreatitis, which can potentiate both conditions, such as abnormal cholesterol metabolism (also involved in bile metabolism), smoking, and dietary factors [25]. An aspect that should not be neglected is the rather high percentage of oncological comorbidities. These can be explained by both etiological (environmental and tobacco) and therapeutic (toxicity of some chemotherapies) factors, as well as aspects related to the status of systemic chronic inflammation. In any case, the comorbidities of patients who develop acute pancreatitis require further studies because their implications for patient survival are important [25,26].

The most common etiology is biliary. Studies have shown an ascending distribution of the disease with age, as well as an association with female sex [27]. The second most common etiology is ethanolic. In the present study, we found that the highest frequency of this condition occurs in cases of severe acute pancreatitis. The same aspect was also observed by Kim DB et al. They observed that most cases of severe acute pancreatitis have alcohol as a central element. Moreover, these cases were associated with necrosis and bacterial infection, increasing the pathogenic inflammatory cascade leading to organ failure [28]. It was observed that there is a dose-dependent relationship in this regard. The consumption of 25 g of alcohol (approximately two drinks per day) is responsible for the development of both acute and chronic pancreatitis. Furthermore, spirits present a much higher risk than wine or beer [29]. It is crucial to remember the measures necessary to prevent severe cases and recurrences in the case of these two etiologies. In the case of biliary etiology, the standard of management provides for cholecystectomy, and in the case of ethanol etiology, abstinence measures are required [30]. Studies regarding the metabolic etiology are controversial. Some have observed that obesity is an independent risk factor for severe acute pancreatitis, while others, especially in the Asian population, have observed that a low body mass index is associated with its development [28]. Li X et al. noted that from the complexity of the metabolic syndrome, fatty liver presents the highest risk for severe acute pancreatitis by maintaining the inflammatory response and increased mortality [31].

Two diagnostic criteria for acute pancreatitis are blood tests and imaging. Blood tests must include serum amylase and lipase [32]. Both should be three times higher than normal, and lipase has the highest affinity. Amylase can also originate from the ovaries, salivary glands, or adipose tissue. Other tests may include urinary trypsinogen-2 or urinary amylase. Both for diagnosis and monitoring, it is necessary to assess the respiratory system (the ratio of the partial pressure of oxygen in arterial blood to the fraction of inspired oxygen), the cardiovascular system (systolic pressure and pH), and the urinary system (serum creatinine) [33].

Important elements in the patient evaluation are represented by triglycerides, which provide additional information about the etiology, and inflammatory markers, which provide information about the pathogenic chain. The meta-analysis performed by Wang Q et al. observed that increased triglyceride levels are associated with increased severity of pancreatitis [34]. These aspects are also supported by Wu Q et al., who noted that increased triglyceride levels during an ICU stay were associated with a higher number of days of hospitalization and with a more severe course of cases. They also observed an association of propofol with increased triglyceridemia [35].

Erythrocyte sedimentation rate (ESR) is an acute-phase inflammatory marker. It has been observed that it is a predictive marker for severe acute pancreatitis. Thus, a value above 60 mm/h at 36 h after admission has a sensitivity of 86% and a specificity of 57% [36]. In the present study, we also observed such stratification at a lower ESR value, with a higher specificity, measured at admission. Another acute-phase inflammation marker with the same ability to distinguish between moderate and severe acute pancreatitis is CRP. Studies indicate a greater affinity for ESR but also a better association with other stratification systems such as CT imaging, Ranson score, or APACHE II [36,37,38]. In the present study, C-reactive protein was associated with CT and APACHE II scores. The severity of inflammation can be calculated by various scores. In the present study, we used the AISI score, which showed associations with the CT score, as well as threshold values for the distinction between moderate and severe acute pancreatitis. The study by Zengin O. et al. noted the effectiveness of this score in clinical practice, both through associations with biological parameters and in assessing the prognosis of patients by predicting days of hospitalization [39]. In contrast to the present study, Araiza-Rodríguez JF et al. observed that the score has a moderate affinity for discriminating between moderate and severe cases [40]. An important aspect to report is the fact that in cases of severe hypertriglyceridemic acute pancreatitis, the AISI score has the lowest predictive value and distinction between the two types of pancreatitis, compared to other scores [41].

A marker of the disease, actively involved in the pathogenic process, is represented by calcium. It provides information on mitochondrial destruction and intra-acinar activation of digestive enzymes [42]. Thus, serum hypocalcemia is independently associated with persistent organ failure, which leads to an increase in mortality rates, reaching 20–50% of cases [43]. In the present study, hypocalcemia represents both a risk factor associated with the development of severe acute pancreatitis and an independent risk factor for mortality in patients with pancreatitis. Hypocalcemia was also associated with inflammatory markers (leukocytes, ESR, CRP), as well as with personal pathological history in the cardiovascular sphere.

Among the interleukins involved in the pathogenic process of pancreatitis, we chose IL-17 because it is less studied in the inflammatory processes of the organ compared to its role in pancreatic tumor processes [44]. Available clinical evidence has demonstrated that serum IL-17 serves as a valuable prognostic indicator in acute pancreatitis, with elevated initial concentrations significantly correlating with disease severity and the development of organ failure [45]. This aspect is explained by the fact that it activates other pro-inflammatory cytokines (TNF-α, IL-1, and IL-6) and produces an influx of neutrophils in the organ. In contrast to IL-6, IL-17 acts as a sentinel of the immune system and is immediately released by damaged epithelium to coordinate a response. In addition, it induces the production and release of IL-6 by stabilizing its mRNA. A positive feedback loop is created in which IL-17 causes the secretion of IL-6, which in turn amplifies and maintains the activity and role of Th17 lymphocytes [45,46]. Vlachos S et al. observed an increase in the first 24 h of IL-17 values with a prediction of 85.3% in cases of acute pancreatitis [47]. Also, IL-17 shows a good association relationship with C-reactive protein and IL-23 [45]. The study by Dongfeng L et al. noted a strong correlation of IL-17 levels depending on the severity of pancreatitis as well as in predicting patient death [48]. In the present study, we showed the role of interleukin in diagnosis (association with amylase), the inflammatory process (association with inflammatory markers), stratification of severity (OR = 1.068), and predicting death (OR = 1.080 in univariate analysis and OR = 1.076 in multivariate analysis). It is worth noting that among the biological markers, it is the only one that, in the multivariate analysis, represents a risk factor for death and, in association with CRP, can stratify pancreatitis. Therefore, assessment of IL-17 levels may provide additional biological information that could aid in patient prognosis and risk assessment. Rather than serving as a stand-alone routine tool, IL-17 requires further investigation to determine its utility as an additional biomarker within multiparametric clinical scoring systems.

From an imaging perspective, the most important predictor remains computed tomography. The scan has the role of providing the Balthazar grade as well as producing an index of severity in conjunction with the percentage of pancreatic necrosis and extra-pancreatic complications [49]. Given the urgency of the condition, along with its accessibility and lower costs, magnetic resonance imaging becomes increasingly important in the late stages. It has a higher contrast resolution in soft tissue, achieving a better characterization of the biliary tree and pancreas, without radiation [50].

Even though the CT score has the highest efficacy, other clinical scores have been developed to assess the severity of pancreatitis, such as APACHE II, Ranson criteria, Glasgow criteria, BISAP, Harmless AP Score, etc. [51]. Among these, APACHE II and BISAP have the highest accuracy in predicting severity and mortality [52]. In the present study, all scoring systems showed statistically significant correlation in the stratification of the two types of pancreatitis studied.

If we look at the days of hospitalization and intensive care, we observe the natural, which consists of longer periods in severe cases. The study conducted by Moran RA et al. highlighted a median of seven hospitalization days in cases of acute pancreatitis [22]. A similar aspect was identified by us. Regarding hospitalization by severity of cases, Koziel D et al. observed median values between 12.5 and 15 days for moderate cases and 5–9 days for severe cases. This inverted balance compared to the present study can be explained by the nature of the study’s age distribution and the increased mortality rate in elderly people with severe acute pancreatitis [23]. Also, Hidalgo NJ et al. observed an average hospitalization rate of 9.38 days, with an average of 13.05 days in intensive care and a mortality rate in patients admitted to intensive care of 35.4% [53]. The 10.34% mortality rate in our moderate acute pancreatitis cohort highlights that, even in the absence of persistent organ failure, baseline cardiovascular history, associated with systemic inflammation and secondary complications, can drastically worsen the prognosis of non-severe patients. Consequently, the mortality rate of patients in the study exceeds the European average (5.7%) and that reported by our country in 2019 (8.6%) [54]. Furthermore, the most recent study published in Romania observed an in-hospital mortality of 5.5% and an intensive care mortality of 38.6% of cases, without mentioning the rates per severity of pancreatitis [2]. Sekulic A et al. identified a mortality rate of 16% in their hospital, which reached 62.5% in those with severe acute pancreatitis [55]. Post-hospitalization mortality, which we could not evaluate, significantly reflects the complexity and severity of the condition. Studies have shown that mortality at 3 months is similar to in-hospital mortality [56].

Aspects that can worsen the in-hospital evolution, also involved in the mortality rate, include complications that can occur. These can be local (edematous interstitial pancreatitis, infected necrosis, pseudocysts, abscesses, etc.), peripancreatic (thrombosis, pseudoaneurysms, or abdominal compartment syndrome), or systemic (pulmonary, cardiac, hepatic, or renal damage) [57,58]. The most common complications were those of fluid effusions, ascites, and pleurisy. CT has the highest accuracy in detecting them. In moderate cases, ascites was detected in 41.4% of cases, and in severe cases, in 37.5% of cases. Pleurisy was detected in 41.4% of moderate cases and in 75% of severe cases [59]. Another study conducted by Bush N et al. noted, analyzing the literature, a frequency of 60–80% of ascites in cases of severe acute pancreatitis. The main causes identified in its occurrence consist of rupture of the pancreatic duct by the inflammatory process, hypoalbuminemia resulting from accentuated catabolism, or portal hypertension caused by thrombosis, especially of the splenic vein [60]. Regarding pleurisy, the meta-analysis conducted by Zeng T et al. noted a frequency of 14% in moderate cases and 67% in severe cases, aspects similar to our study. It also observed that pleurisy has a major influence on the death of patients [61].

A major infectious complication is represented by a pancreatic abscess. A pancreatic abscess forms as a result of the progressive liquefaction of necrotic pancreatic parenchyma and adjacent tissue. Some abscesses can form as a superinfection of peripancreatic fluid collections [62]. Their occurrence has a frequency between 3% and 5.45%, and the mortality rate can vary between 10% and 30% [63]. Complications such as infected necrosis, which occur in a third of cases, represent a late event in acute pancreatitis. However, a quarter of cases occur in the first two weeks. The persistence of the systemic inflammatory response syndrome is responsible for the occurrence of this complication [64]. The study conducted by Chen HZ et al. identified a frequency of 40.5% of infected pancreatic necrosis and a mortality of 13.8% in a group of moderate and severe acute pancreatitis [65]. The mortality rate of these cases can reach 50–80% [65,66].

Other major complications consist of organ failure in the first weeks of hospitalization. These usually have a median duration of one week. The respiratory tract (92%), cardiovascular (82%), and renal (44%) systems are affected [67]. Acute respiratory distress syndrome often manifests pulmonary involvement. Its frequency is between 2.63% and 92%, with mortality between 17% and 87% [68]. The most frequent cardiovascular dysfunctions, complications of acute pancreatitis, are represented by rhythm disorders (possibly caused by fluid resuscitation), myocardial damage (caused by oxidative stress and systemic inflammatory response syndrome), or chronic heart failure (caused by volume overload). A large part of these patients require vasoactive treatment. In the present study, especially patients with severe acute pancreatitis required such treatment. The study by Shi H et al. showed a 31.1% use of vasoactive treatment in intensive care units. However, they observed that their early use increases in-hospital mortality [69]. Furthermore, the study by Shafiq F et al. observed an 80% use of them in cases admitted to intensive care [70]. All these complications, which are based on hypoxic and circulatory changes, can lead to the development of acute kidney injury. As a rule, the condition occurs after the failure of other organs; in 8.9% of cases, the kidney was the first affected. The mortality of these patients is between 25% and 75% [71].

The limitations of this study are the retrospective nature, the absence of data on patient habits such as smoking and alcohol consumption, the exact value of the body mass index, and a small sample size. This limited cohort increases the risk of overfitting and statistical inflation from multiple testing, which can lead to unstable estimates or false positive results in the multiple statistical analyses and multivariable logistic regressions performed. The absence of mild cases inherently restricts the clinical interpretation of IL-17, introducing a risk of circular severity classification, as we could not determine whether it distinguishes severe disease from the general population with acute pancreatitis or simply separates two already high-risk groups. In addition, the study design is susceptible to potential selection bias, unmeasured treatment confounding, and residual or unmeasured confounders, as certain clinical variables or nonstandardized emergency interventions could not be fully controlled. Crucially, the proposed serum IL-17 thresholds lack serial sampling to assess temporal dynamics, include post-baseline predictors (such as complications and ICU stay duration) within the mortality analyses, and lack external validation in an independent, multicenter cohort. In contrast, the strengths of this study consist of the identification of potential predictors and factors associated with mortality (cardiovascular and renal comorbidities, metabolic etiology, CRP value, IL-17, and hypocalcemia). Serum IL-17 levels also demonstrated a significant association within the inflammatory cascade through correlations with specific biological markers, established risk scores, and the stratification of threshold values between moderate and severe pancreatitis (Figure 2). Therefore, these results should be interpreted with caution, and external validation of the identified thresholds on much larger, independent cohorts is strictly necessary to determine their true clinical utility.

## 5. Conclusions

In the present study, we identified parameters that are associated with existing scoring systems in the severity stratification of moderate and severe acute pancreatitis, including leukocytosis, CRP, IL-17, and hypocalcemia. In addition, factors such as comorbidities (cardiovascular and renal), metabolic etiology, and laboratory parameters (CRP, IL-17, and hypocalcemia) emerged as potential predictors of mortality, while duration of intensive care unit stay and complications functioned as factors associated with mortality. Finally, reflecting the observational nature of this study, IL-17 may provide valuable information on the progression of moderate and severe acute pancreatitis. Its positive correlation with established scoring systems and inflammatory markers highlights its potential utility in severity stratification, while the identified cut-off values must be interpreted as preliminary rather than validated predictors of mortality. Ultimately, prospective studies with larger cohorts and external validation are strictly required before the clinical utility or predictive performance of IL-17 and the proposed thresholds can be established.

## Figures and Tables

**Figure 1:**
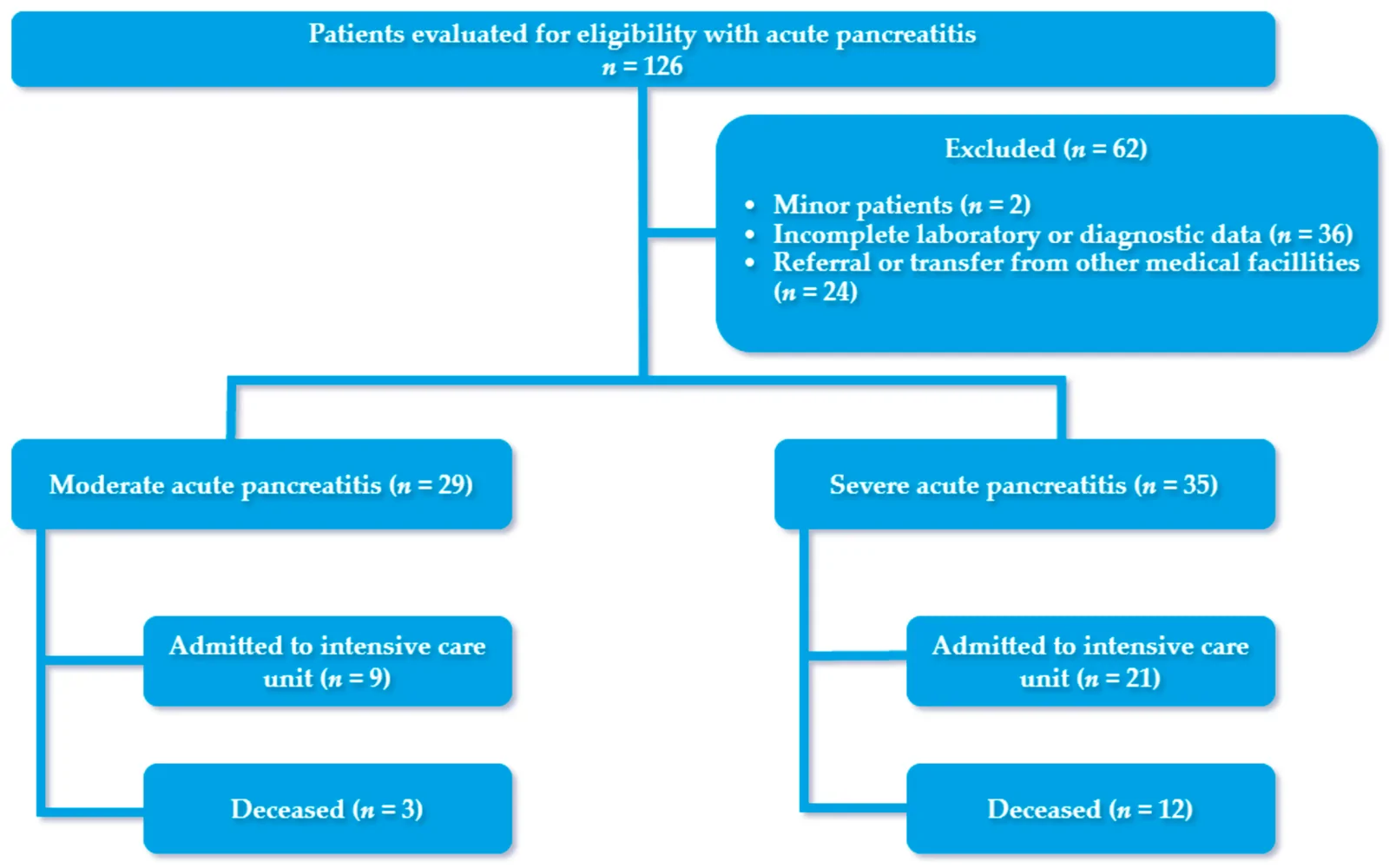
Flowchart of patient inclusion and exclusion criteria according to the severity of acute pancreatitis.

**Figure 2:**
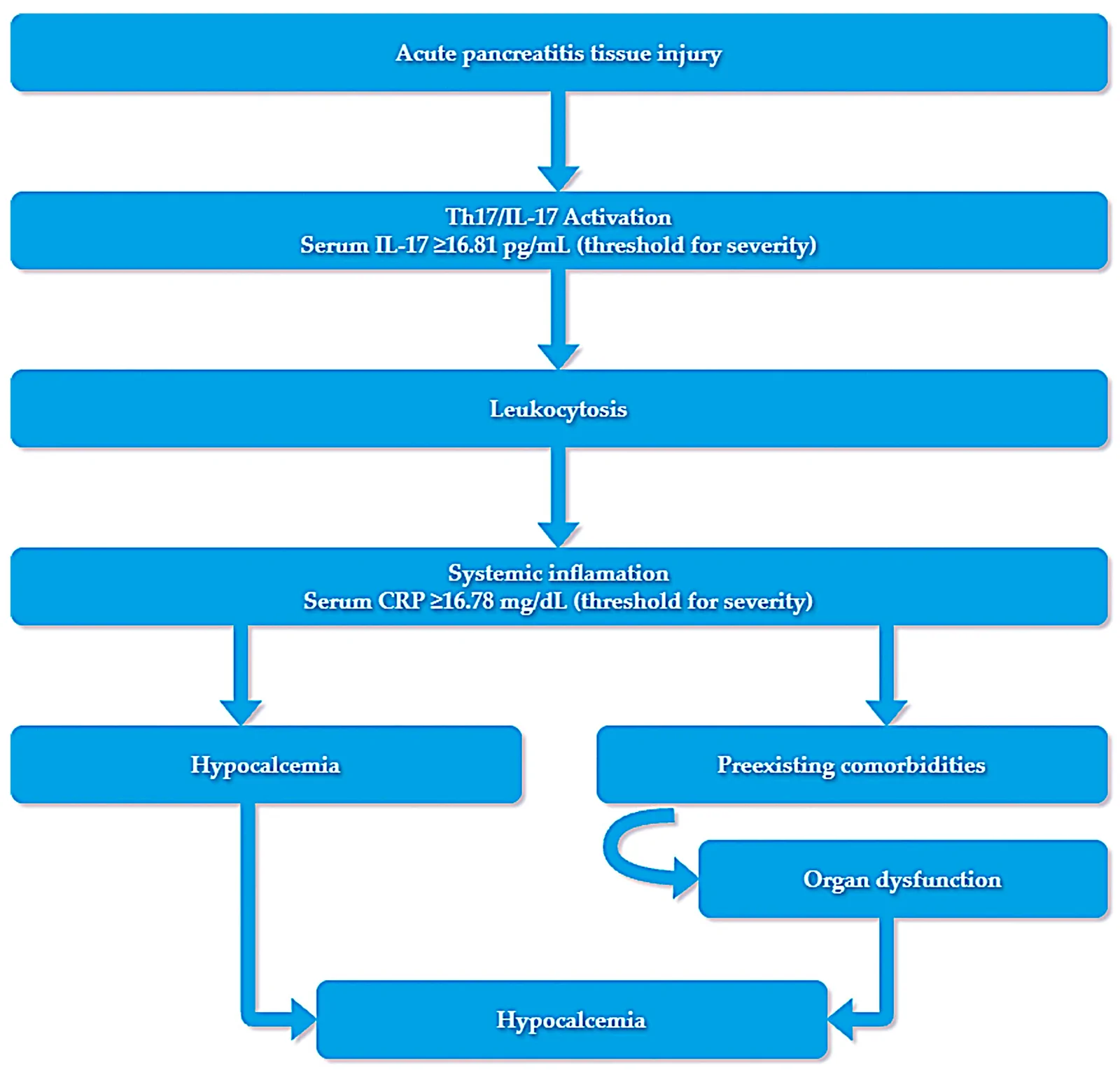
Conceptual summary of the proposed pathophysiological cascade. This flowchart represents a conceptual framework that illustrates observed statistical associations, rather than definitive causal pathways, within the study cohort.

**Table 1:** Comparative clinical–demographic aspects of the study group.

	Moderate Acute Pancreatitis	Severe Acute Pancreatitis	*p*-Value
Gender, n (%) Female: Male	13 (44.83):16 (55.17) 1:1.23	10 (28.57):25 (71.43) 1:2.5	0.201
Age, median (IQR)	55 (42–75)	64 (51–76)	0.297
Average, n (%) Rural: Urban	17 (58.62):12 (41.38) 1.42:1	9 (25.71):26 (74.29) 1:2.89	0.011
Personal history of respiratory pathology, n (%)	2 (6.90)	3 (8.57)	0.590
Personal history of cardiovascular disease, n (%)	14 (48.28)	15 (42.86)	0.802
Personal history of digestive pathologies, n (%)	17 (58.62)	23 (65.71)	0.611
Diabetes mellitus, n (%)	7 (24.14)	8 (22.86)	0.586
Personal history of renal pathology, n (%)	1 (3.45)	5 (14.29)	0.209
Personal history of neurological pathology, n (%)	5 (17.24)	5 (14.29)	0.505
Personal history of oncological pathologies, n (%)	3 (10.34)	5 (14.29)	0.719

**Table 2:** Univariate and multivariate analyses of risk factors for death in patients with acute pancreatitis.

Death	Univariate Analysis			Multivariate Analysis		
	OR	CI95	*p*-Value	OR	CI95	*p*-Value
Personal history of cardiovascular disease	7.529	1.866–30.387	0.005	4.667	1.063–20.494	0.041
Personal history of renal pathology	24.000	2.523–228.284	0.006	11.429	1.119–116.700	0.040

**Table 3:** Etiology of pancreatitis in the studied group.

	Moderate Acute Pancreatitis	Severe Acute Pancreatitis	*p*-Value
Biliary etiology, n (%)	12 (41.38)	14 (40)	0.556
Ethanol etiology, n (%)	9 (31.03)	16 (45.71)	0.305
Metabolic etiology, n (%)	2 (6.90)	7 (20)	0.166
Post-interventional etiology, n (%)	6 (20.69)	2 (5.71)	0.127

**Table 4:** Comparative biological parameters in acute pancreatitis.

	Moderate Acute Pancreatitis	Severe Acute Pancreatitis	*p*-Value
Amylase increased, n (%)	23 (79.31)	31 (88.57)	0.491
Serum amylase U/L, median (IQR)	499.95 (136.59–1583.99)	823 (300–3531.6)	0.356
Elevated lipase, n (%)	26 (89.66)	32 (91.43)	0.569
Serum lipase U/L, median (IQR)	866.90 (319–5918)	1379.46 (300–2194.8)	0.995
Leukocytosis, n (%)	15 (51.72)	28 (80)	0.031
Leukocyte count ×10^3^ µL, median (IQR)	11 (9.4–13.49)	19.45 (14.62–25.98)	<0.001
ESR mm/h, median (IQR)	34.46 (11.35–49.15)	50.08 (29.5–72.15)	0.012
CRP mg/dL, median (IQR)	9.8 (4.33–14.85)	24.4 (14.08–30)	<0.001
AISI, median (IQR)	1120 (607.5–1642)	4216 (2487–6384)	<0.001
IL-17 pg/mL, median (IQR)	16.63 (11.43–20.82)	19.03 (16.87–25.56)	0.038
pH, median (IQR)	7.41 (7.37–7.46)	7.37 (7.26–7.41)	0.174
Bicarbonate mmol/L, median (IQR)	24.60 (19.20–26.70)	18 (14–23.90)	0.099
Lactate mmol/L, median (IQR)	1.85 (1.01–3.39)	3.01 (2.23–4.37)	0.089
Low calcium, n (%)	12 (41.38)	25 (71.43)	0.022
Blood sugar:			0.842
Low, n (%)	3 (10.34)	3 (8.57)	
High, n (%)	4 (13.79)	7 (20)	
Elevated triglycerides, n (%)	3 (10.34)	5 (14.29)	0.719

**Table 5:** Univariate and multivariate analyses on the predictive risk factors of severe acute pancreatitis.

Severe Acute Pancreatitis	Univariate Analysis			Multivariate Analysis		
	OR	CI95	*p*-Value	OR	CI95	*p*-Value
Leukocytosis	3.733	1.239–11.249	0.019	2.132	0.510–8.916	0.300
CRP	1.136	1.064–1.213	<0.001	1.149	1.061–1.245	0.001
IL-17	1.068	1.001–1.141	0.048	1.092	1.003–1.189	0.043
Hypocalcemia	3.542	1.250–10.031	0.017	1.044	0.260–4.190	0.952

**Table 6:** Univariate and multivariate analyses on risk factors for death in patients with acute pancreatitis.

Death	Univariate Analysis			Multivariate Analysis		
	OR	CI95	*p*-Value	OR	CI95	*p*-Value
CRP	1.051	1.002–1.103	0.042	1.036	0.981–1.093	0.206
IL-17	1.080	1.011–1.154	0.022	1.076	1.007–1.149	0.030
Hypocalcemia	6.771	1.380–33.222	0.018	4.597	0.813–26.007	0.084

**Table 7:** Central summary of candidate biomarkers for the prediction and severity stratification of moderate and severe acute pancreatitis.

Biomarker	Moderate Acute Pancreatitis	Severe Acute Pancreatitis	Univariable OR (CI95)	Multivariable OR (CI95)	AUC (CI95)	Cut-Off	Sensitivity (%)	Specificity (%)	Significant After Adjustment?
IL-17 ^a^	16.63 (11.43–20.82)	19.03 (16.87–25.56)	1.068 (1.001–1.141)	1.092 (1.003–1.189)	0.649 (0.513–0.785)	≥16.81	77.1%	55.2%	Yes (*p* = 0.030)
CRP ^a^	9.8 (4.33–14.85)	24.4 (14.08–30)	1.136 (1.064–1.213)	1.149 (1.061–1.245)	0.817 (0.712–0.922)	≥16.78	68.6%	82.8%	Yes (*p* = 0.043)
Leukocytosis ^b^	51.72	80	3.733 (1.239–11.249)	2.132 (0.510–8.916)	— ^c^	— ^c^	— ^c^	— ^c^	No (*p* > 0.050)
Hypocalcemia ^b^	41.38	71.43	3.542 (1.250–10.031)	1.044 (0.260–4.190)	— ^c^	— ^c^	— ^c^	— ^c^	No (*p* > 0.050)

^a^ Reported as median (interquartile range) for continuous variables. ^b^ Reported as percentage (%) for nominal variables. ^c^ ROC analysis is mathematically not applicable (—) for nominal variables.

**Table 8:** Comparative aspects of diagnostic scores.

	Moderate Acute Pancreatitis	Severe Acute Pancreatitis	*p*-Value
CT score			<0.001
A–B, n (%)	10 (34.48)	0 (0)	
C, n (%)	19 (65.52)	0 (0)	
D, n (%)	0 (0)	10 (28.57)	
E, n (%)	0 (0)	25 (71.43)	
RANSON, median (IQR)	3 (1–5)	5 (3–7)	<0.001
IMRIE–Glasgow, median (IQR)	2 (1–3)	4 (2–5)	0.001
BISAP, median (IQR)	1 (1–2)	3 (2–4)	<0.001
SOFA, median (IQR)	3 (2–4)	4 (2–8)	0.040
APACHE, median (IQR)	7 (2.5–10)	11 (5–19)	0.002
SAPS3, median (IQR)	43 (35–53.5)	53 (43–69)	0.008

**Table 9:** Comparative aspects regarding the intrahospital management of the patient group.

	Moderate Acute Pancreatitis	Severe Acute Pancreatitis	*p*-Value
Outcomes			
Hospitalization days, median (IQR)	6 (4–10)	9 (5–12)	0.474
ICU days, median (IQR)	0 (0–1.5)	2 (0–7)	0.077
Death, n (%)	3 (10.34)	12 (34.29)	0.037
Local Complications			
Abscess-type complications, n (%)	1 (3.45)	8 (22.86)	0.033
Infected necrosis-type complications, n (%)	0 (0)	8 (22.86)	0.006
Ascites-type complications, n (%)	4 (13.79)	26 (74.29)	<0.001
Systemic complications and dysfunctions			
Pleurisy-type complications, n (%)	4 (13.79)	21 (60)	<0.001
Pulmonary dysfunction, n (%)	2 (6.90)	12 (34.29)	0.013
Acute respiratory distress syndrome, n (%)	1 (3.45)	9 (25.71)	0.017
Cardiovascular dysfunction, n (%)	3 (10.34)	14 (40)	0.010
Need for vasoactive support, n (%)	2 (6.90)	14 (40)	0.003
Renal dysfunction, n (%)	4 (13.79)	14 (40)	0.026

**Table 10:** Univariate and multivariate analyses on risk factors for death in patients with acute pancreatitis.

Death	Univariate Analysis			Multivariate Analysis		
	OR	CI95	*p*-Value	OR	CI95	*p*-Value
ICU Days	1.343	1.119–1.611	0.002	1.121	0.947–1.327	0.183
Abscess-type complications	10.222	2.150–48.606	0.003	5.405	0.115–253.446	0.390
Infected necrosis-type complications	7.667	1.569–37.458	0.012	4.863	0.087–271.623	0.441
Ascites-type complications	4.342	1.207–15.626	0.025	9.444	0.527–169.061	0.127
Pleurisy-type complications	4.533	1.320–15.564	0.016	2.272	0.175–29.443	0.530
Acute respiratory distress syndrome	72.000	7.715–671.944	0.000	18.957	0.683–526.146	0.083
Need vasoactive support	45.000	8.845–228.931	0.000	9.567	0.617–148.439	0.106

**Table 11:** Univariate and multivariate analyses on risk factors for death in patients with acute pancreatitis.

Death	Univariate Analysis			Multivariate Analysis		
	OR	CI95	*p*-Value	OR	CI95	*p*-Value
Pulmonary dysfunction	94.000	14.085–627.328	<0.001	2.256	0.088–57.981	0.623
Cardiovascular dysfunction	73.125	12.014–445.082	<0.001	21.312	1.047–433.953	0.047
Renal dysfunction	28.667	6.229–131.925	<0.001	9.148	0.989–84.616	0.051

## Data Availability

The original contributions presented in this study are included in the article. Further inquiries can be directed to the corresponding authors.

## Supplementary Materials

The following supporting information can be downloaded at: https://www.mdpi.com/article/10.3390/medsci14060600/s1, Supplementary Tables S1–S6. Supplementary Table S1. Multivariate analysis of risk factors for death in patients with acute pancreatitis. Supplementary Table S2. Multivariate analysis of risk factors for death in patients with acute pancreatitis. Supplementary Table S3. Multivariate analysis of risk factors for death in patients with acute pancreatitis. Supplementary Table S4. Multivariate analysis of risk factors for death in patients with acute pancreatitis. Supplementary Table S5. Multivariate analysis of risk factors for death in patients with acute pancreatitis. Supplementary Table S6. Multivariate analysis of risk factors for death in patients with acute pancreatitis.
