# Supplementary material for: The Role of Interleukin 17 in Association with Systemic Parameters in Severity Stratification and Mortality Prediction in Moderate and Severe Acute Pancreatitis
Source: Med Sci (Basel). 2026 Sep 23;14(6):600. doi: 10.3390/medsci14060600 (PMC13706923; doi:10.3390/medsci14060600)
Supplement: Supplementary file 1 [file medsci-14-00600-s001.zip › medsci-4552468-supplementary.pdf]

Supplementary Table S1. Multivariate analysis of risk factors for death in patients with acute pancreatitis.

Variable	B	S.E.	Wald	df	Sig. (*p*-Value)	Exp(B) (Adjusted OR)	95% C.I. for EXP(B) (Lower)	95% C.I. for EXP(B) (Upper)
Cardiovascular Dysfunction	4.259	0.938	20.614	1	<0.001	70.747	11.252	444.832
Age (Years)	0.028	0.028	0.944	1	0.331	1.028	0.972	1.087

Supplementary Table S2. Multivariate analysis of risk factors for death in patients with acute pancreatitis.

Variable	B	S.E.	Wald	df	Sig. (*p*-Value)	Exp(B) (aOR)	95% C.I. for EXP(B) (Lower)	95% C.I. for EXP(B) (Upper)
Cardiovascular Dysfunction	4.160	0.957	18.903	1	<0.001	64.097	9.825	418.163
Disease Severity (Severe AP)	0.421	1.013	0.173	1	0.677	1.524	0.209	11.088

Supplementary Table S3. Multivariate analysis of risk factors for death in patients with acute pancreatitis.

Variable	B	S.E.	Wald	df	Sig. (*p*-Value)	Exp(B) (aOR)	95% C.I. for EXP(B) (Lower)	95% C.I. for EXP(B) (Upper)
Cardiovascular Dysfunction	4.150	0.977	18.054	1	<0.001	63.452	9.355	430.392
Age (Years)	0.027	0.028	0.894	1	0.344	1.027	0.972	1.086
Disease Severity (Severe AP)	0.347	1.024	0.115	1	0.735	1.415	0.190	10.524

Supplementary Table S4. Multivariate analysis of risk factors for death in patients with acute pancreatitis.

Variable	B	S.E.	Wald	df	Sig. (*p*-Value)	Exp(B) (aOR)	95% C.I. for EXP(B) (Lower)	95% C.I. for EXP(B) (Upper)
Renal Dysfunction	3.257	0.786	17.174	1	<0.001	25.969	5.565	121.177
Age (Years)	0.017	0.024	0.494	1	0.482	1.017	0.971	1.065

Supplementary Table S5. Multivariate analysis of risk factors for death in patients with acute pancreatitis.

Variable	B	S.E.	Wald	df	Sig. (*p*-Value)	Exp(B) (aOR)	95% C.I. for EXP(B) (Lower)	95% C.I. for EXP(B) (Upper)
Renal Dysfunction	3.171	0.791	16.058	1	<0.001	23.826	5.053	112.352
Disease Severity (Severe AP)	0.921	0.855	1.161	1	0.281	2.512	0.470	13.414

Supplementary Table S6. Multivariate analysis of risk factors for death in patients with acute pancreatitis.

Variable	B	S.E.	Wald	df	Sig. (*p*-Value)	Exp(B) (aOR)	95% C.I. for EXP(B) (Lower)	95% C.I. for EXP(B) (Upper)
Renal Dysfunction	3.100	0.801	14.963	1	<0.001	22.196	4.615	106.762
Age (Years)	0.012	0.024	0.250	1	0.617	1.012	0.965	1.061
Disease Severity (Severe AP)	0.832	0.868	0.920	1	0.338	2.299	0.420	12.599
